# Crown and diameter structure of pure *Pinus massoniana* Lamb. forest in Hunan province, China

**DOI:** 10.1515/biol-2022-0574

**Published:** 2023-03-02

**Authors:** Siwen Su, Nan Deng, Fengfeng Ma, Qingan Song, Yuxin Tian

**Affiliations:** Faculty of Life Science and Technology, Central South University of Forestry and Technology, Changsha 410004, Hunan Province, China; Hunan Academy of Forestry, Changsha 410004, Hunan Province, China; Hunan Cili Forest Ecosystem State Research Station, Cili, 427200, Hunan Province, China

**Keywords:** diameter at breast height, vertical structure, gradient boosting model, site conditions

## Abstract

Non-spatial structure of forest is an important aspect for harvesting regimes, silvicultural treatments, and ecosystem service provisions. In this pursuit, the present research envisaged the measurement of the crown and diameter structure of *Pinus massoniana* Lamb. Specifically, the forests were assessed with a range of nine cities in Hunan Province, China. The gradient boosting model was used to quantify the contribution of seven drivers of the diameter at breast height (DBH) diversity. Moreover, the relationship between the crown structure and DBH/tree height was explored using TSTRAT and path analysis. The Anderson–Darling test results indicated that DBH distributions of nine cities did not occur from the same population, the maturing diameter distribution was the main type among the cities. Slope direction was identified as the most impacted factor affecting the DBH diversity, followed by landform and stand density. The vertical stratification indicated a simple vertical structure, and the relationship between the DBH/tree height and crown structure changed in different stages, which reflected the competition mechanism and adaption strategy in the forest. Our study summarized the diameter and crown structure of pure *P. massoniana* forest in Hunan province, which can provide valuable information in the forest management, planning, and valuation of ecosystem services.

## 1 Introduction

Forest is an ecosystem that functions with a self-growth regulation, and its structure affects the biomass and productivity [1,2]. The structure of the forest affects the biodiversity, biomass production, along with the habitat function [3]. A reasonable adjustment of the forest structure is conducive to its ecological function [4]. Forest structure consists of vertical and horizontal components and consists of the species composition, size, and distribution of trees, shrubs, and ground cover vegetation [5,6]. Vertical stand structure often refers to the layering of tree crowns, while the horizontal structure mostly represents the distribution of the diameter and spatial patterns of the tree species [7]. So far, many models have been applied in different tree species to describe the forest structures [8–10]. Forest structure diversity can reflect the complexity of stands, which can benefit the forest monitoring, formulating policy, management, and assessing the ecosystem services [11,12]. The assessment of structure diversity has been performed based on the variety of variables including the species number, tree size, foliage height, coarse woody debris, etc. [5,13]. Among them, structural diversity can be assessed by the distribution of diameter of a population of trees. Previous studies show that the diameter distributions can be classified into three types as youthful distribution, maturing distribution, and mature distribution that is important in explaining the natural progression of the distribution of diameter [11,14,15]. Diameter distribution varies with the management, tree species, stand dynamics, and disturbance regimes [16]. Many factors such as altitude, slope direction, soil type, and soil nutrient affect the diameter at breast height (DBH) that were used for type site division, but the quantitative study of these effects is limited.

The vertical structure is mostly represented with the distribution of tree height and crown, and many indexes have been proposed to characterize the vertical structure [17–19]. Additionally, the tree crowns determine the light interception, gas exchange and water through photosynthesis and evapotranspiration, which influence many aspects of the ecosystem [20]. Thus, it plays a very important role in the vertical structure analysis. Tree crown allometry describes the scaling relationships between the crown dimensions and more easily measurable variable such as stem diameter, which is widely used in the quantification of the ecosystem function and determination of the tree spacing [20,21]. The tree height and DBH are considered as the best indicators in crown prediction [21,22]. In the modeling process, many approaches like the mixed effect model, spatial statistics model, and multiple regression models have been used to develop appropriate and valuable models [23,24], but the effects and application are limited.

Masson pine (*Pinus massoniana* Lamb.) is a major coniferous tree species that is widely distributed in the subtropical forests of South China [25]. In Hunan province, the Masson pine is an important source of timber and wood pulp, and most pine forests are artificial pure forests. Previous studies of Masson pine are limited to a single city or subset of the tree population. They provide valuable information for the practical management, but fail to provide a comprehensive assessment of the diameter distribution or crown structure in the Hunan province. The present study was aimed to assess the Masson pine population in Hunan province in terms of tree height, stem diameter at DBH, and crown structure, with the following objectives: (1) assessment of the DBH structure of trees in each city, (2) quantifying the impact of common stand dynamics factors on DBH diversity at a large scale, and (3) develop path model to provide a theoretical basis for establishing a better crown size prediction model at large scale. Additionally, based on our analysis, some suggestions are provided in regards to the forest plantation.

## 2 Materials and methods

### 2.1 Data sources

**Figure 1:**
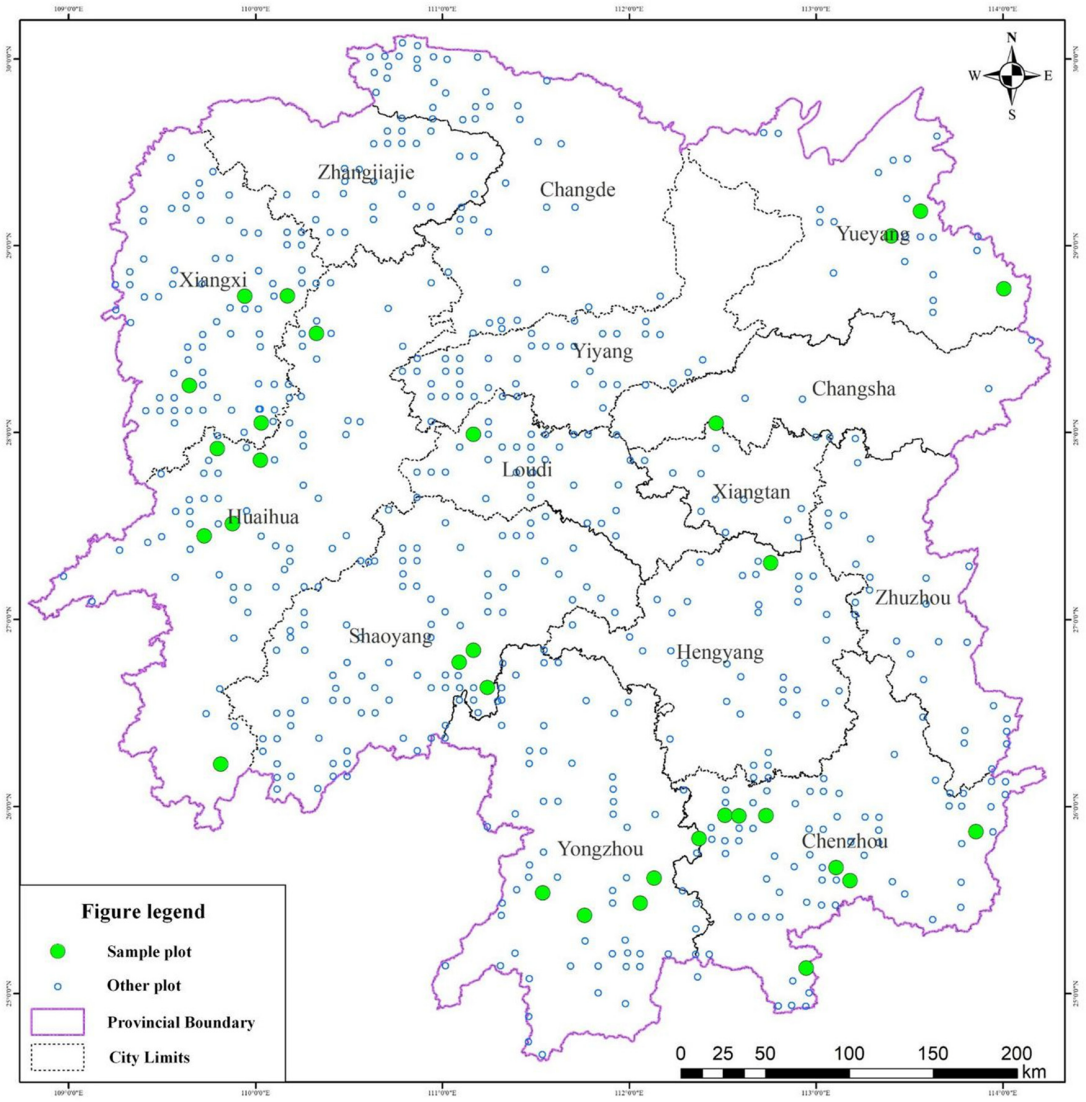
Locations of 31 plots.

An ecological forest has the function of maintaining the ecological balance and protecting the biodiversity. The ecological forest is the main forest resource that covers 36.65% of the Hunan province, and deforestation is not allowed in this ecological forest. The forest survey data were obtained from the forest fixed sample plot investigation database of Hunan ecological forests (updated in 2019). A total of 683 fixed sample plots in this database were set by equal space in the area of ecological forest, and the space was calculated according to the total forest area in the geographical information system software. The size of plot was 25 m (vertically to the counter line) × 40 m (parallel to contour line). The crown width (average of two crown diameters perpendicular to each other), tree height, and DBH of each tree were measured. The crown length was calculated as the height from the first living branch to the top. Additionally, the stand dynamics factors such as altitude, slope position, and stand density of each plot were also measured. In this study, 31 pure *P. massoniana* forest plots were selected, and a total of 3,633 trees were sampled that distributed among nine cites of the Hunan province (Table 1, Figure 1). The distribution range of GPS data within the study sites was 109.6465°–114.0073°E, 25.1357°–30.0865°N. The altitude of all plots ranged from 105 to 1,250 m. The forests’ age ranged from 8 to 51 years, the average DBH on all trees in the plots ranged from 6.7 to 24.8 cm, and the height of the trees in all plots ranged from 5.4 to 20 m.

**Table 1:** Information of 31 pure *P. massoniana* forest plots

City	Sampled plots	Sampled trees (number)
Chenzhou	8	1,123
Hengyang	1	80
Huaihua	6	729
Loudi	1	28
Shaoyang	3	218
Xiangtan	1	73
Xiangxi	4	434
Yongzhou	4	467
Yueyang	3	481

### 2.2 Horizontal structure analysis

The DBH, crown length, and tree height of the forest are the basic parameters in a stage of certain age, which reflects the growth of the forest. In this study, all the *P. massoniana* plots were divided into four stages of age, first: young aged (≤20), middle aged (21–30), near mature (31–40), and mature (41–60), where the age in years is presented in parentheses. The DBH, tree height, crown width, and crown length of four stages were analyzed and compared. Wilcoxon test for differences between age stages was conducted.

To assess the DBH structure of trees in the whole area and nine cities, the plots were grouped according to the city distribution. Anderson–Darling (AD) *k*-Sample test was initially used to test the null hypothesis between the cities that all DBH distributions occurred from a common population. The AD test is a robust and proven method to test the differences among the DBH distributions [26]. The *p*-value <0.05 indicated that the samples were not from the same population. The DBH was divided into following classes: ≤5, 5–8, 8–12, 12–16, 16–20, 20–24, 24–28, 28–32, 32–36, and 36–40 cm. The Shannon–Wiener index was used to calculate the structural diversity index from the proportional abundance of trees in different DBH classes [11,27]:

(1) H=-\mathop{\sum }\limits_{i=1}^{{n}}{p}_{i}\hspace{.25em}\mathrm{ln}({p}_{i}),]

where *p*_*i*_ is the proportion of trees in the *i*th diameter class and *n* is the number of diameter classes (*n* = 10 for all cities). The Shannon–Wiener index was calculated for each city and plot.

### 2.3 Identification of driving factors for the DBH diversity

From the selected plots, five stand dynamics factors were extracted from the database to estimate their contribution to the DBH diversity. These factors included the altitude, stand density (number of trees in 1,000 m^2^), landform, slope direction, and soil type (red soil, lateritic red soil, and yellow soil). Landforms were divided into three types in this study: hill (altitude range from 200 to 500 m), low-mountain (altitude range from 500 to 1,000 m), and mid-mountain (altitude range from 1,000 to 3,500 m). Additionally, the average temperature and average precipitation of each plot was gained from the world climate database (WorldClim: http://www.worldclim.org/). The above factors were considered as the basic components that reflected the potential forest productivity according to the Chinese National Standard-Technical regulations as an inventory for the forest management planning and design (GB/T 26424-2010). We quantified the relative contribution of these seven drivers to the DBH diversity of all plots using the gradient boosting model (GBM) [28], based on the Shannon–Wiener index of each plot. This model can fit the nonlinear relationship continuously between diversity and factors, and its flexibility, explanatory variable selection and cross-validation approach offer an advantage in the ecology studies [29,30]. Additionally, the marginal plots were constructed, which reflected the influence from one predictor variable, when the other predictor variables were fixed.

### 2.4 Vertical structure analysis

The TSTRAT algorithm developed by Latham et al. [17] was used to assess the vertical structure of forest. The algorithm determines a vertical height cut-off point and assigns trees to vertical strata based on tree heights and crown length layers. The formula is shown in the following equation:

(2) \text{CPS}=0.4\text{CL}+\text{HBLC,}]

where CPS represents the cut-off height value, HBLC represents the height-to-base of the live crown, and CL represents the crown length. The coefficient 0.4 arises from the consideration that the competition to stay in an advantageous position for the acquisition of light was the greatest in the top 60% of the tree crown. The vertical strata of each plot were calculated by the TSTRAT algorithm to assess the vertical structure of the whole population.

The tree height and DBH were considered as the best indicators in crown prediction [21,22]. But in practical application, due to site conditions, management measures, human activities, and other factors, the relationship between the crown and other factors is complex and may be nonlinear. The traditional correlation analysis only describes the overall relationship between two or more characteristics. However, the path analysis method developed by Wright [31] and Bhatt [32] to study the direct and indirect relationships between multiple independent variables and a dependent variable is useful in determining the contribution of component variables to a character [33]. Hence, in this study, the path analysis was used to analyze the effects of height and DBH on crown width and live crown ratio under different age classes. The live crown ratio was calculated as per the following equation:

(3) R=L/H,]

where *L* represents the crown length (height from the first living branch to the top) and *H* represents the tree height.

The path coefficients were calculated as follows:

(4) \text{Correlation coefficient}:\hspace{.5em}{r}_{ij}=C{v}_{ij}\text{/}\sqrt{{{\sigma }}_{i}^{2}{{\sigma }}_{j}^{2}},]

(5) \text{Direct path coefficient}:\hspace{.5em}{P}_{yi}={b}_{i}{s}_{i}\text{/}{s}_{y},]

(6) \text{Indirect path coefficient}:\hspace{.5em}{P}_{yij}={r}_{ij}{P}_{yi}.]

where *Cv*_*ij*_ represents the covariance of *i* and *j*, {\sigma }_{i}^{2}] and {\sigma }_{j}^{2}] represent the variance of *i* and *j*, *b*_*i*_ represents the partial regression coefficient of *i* and *j*, *s*_*i*_ and *s*_*y*_ are the standard deviations of *i* and *y*, and *y* represents the crown radius and live crown ratio.

All the data analysis and graphics were conducted by using R Core Team, ggplot2 [34], pastecs [35], SEM [36], lattice [37], and gbm [38] software packages.

## 3 Results

### 3.1 DBH, height, and crown distributions of different age stages

**Figure 2:**
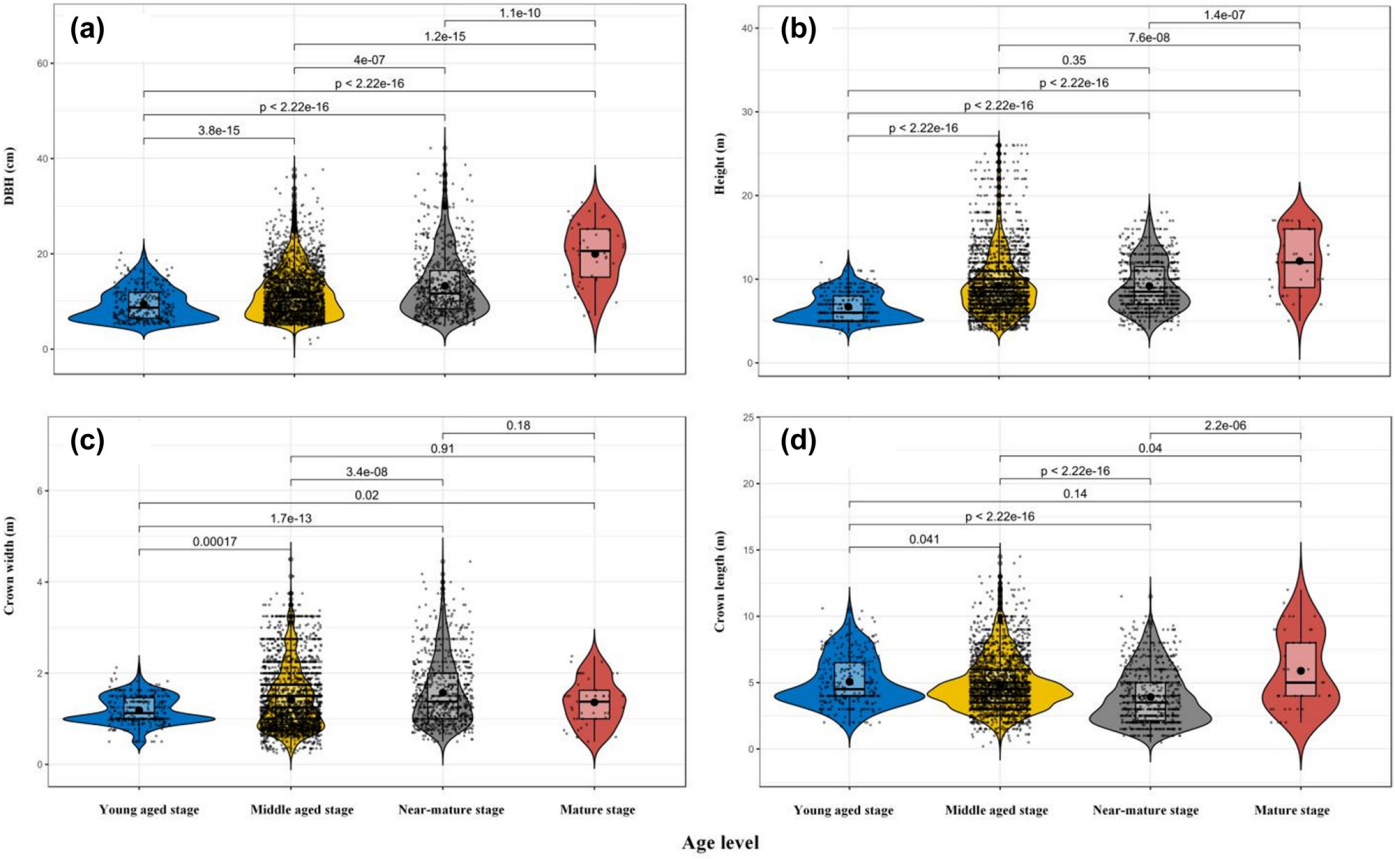
Violin plot of DBH (a), height (b), crown width (c), and crown length (d) (values represent the Wilcoxon test between the stages of age).

The DBH distribution of four age stages is shown in Figure 2a. The average DBH value increased with the age stage (*p* < 0.05). The DBH distribution at the succession stages appeared to be inclined to the right side, except that of the mature stage. The average DBH from young to mature stage were 8.73, 12.19, 14.4, and 21.75 cm, respectively. The height of young aged stage was significantly lower than that of the other stages (*p* < 0.05). Middle aged and near-mature stage showed no significant difference, and the distributions of the young aged, near-mature, and mature stages were multimodal (Figure 2b). The crown width of the young aged, middle aged, and near-mature stages showed statistically different variables between each two stages. The distributions were multimodal except for the near-mature stage (Figure 2c). The crown length of the young aged and mature stage was significantly higher than that of the other stages, and the distribution appeared to be inclined to the right side, except in that of the mature stage (Figure 2d).

### 3.2 DBH structure of trees in each city

It was observed that the AD *k*-Sample test (*p* < 2.2 × 10^−16^) rejected the null hypothesis, i.e., the diameter distributions of nine cities did not occur from the same population. The skewness and kurtosis of the DBH distribution in each city were calculated (Table 2). The skewness of Yongzhou was highest that indicated the greatest degree of deviation (1.6807), and the distribution was right-skewed except in case of Loudi. The kurtosis indicated that the distribution was steep compared to the normal distribution except for the cities Hengyang and Loudi, while Xiangtan and Yongzhou had the greatest degree of deviation (4.111).

**Table 2:** Normal distribution test and diversity of DBH distribution in nine cities

City	Skewness	Kurtosis	Shannon–Wiener index
Chenzhou	1.1024	1.7682	1.323
Hengyang	0.4374	−0.0692	1.666
Huaihua	0.9008	1.1986	1.564
Loudi	−0.2733	−1.0658	1.846
Shaoyang	0.7673	0.3982	1.689
Xiangtan	0.0028	−0.7865	1.762
Xiangxi	0.8231	0.6559	1.895
Yongzhou	1.6807	4.1112	1.508
Yueyang	0.9925	0.9834	1.449

**Figure 3:**
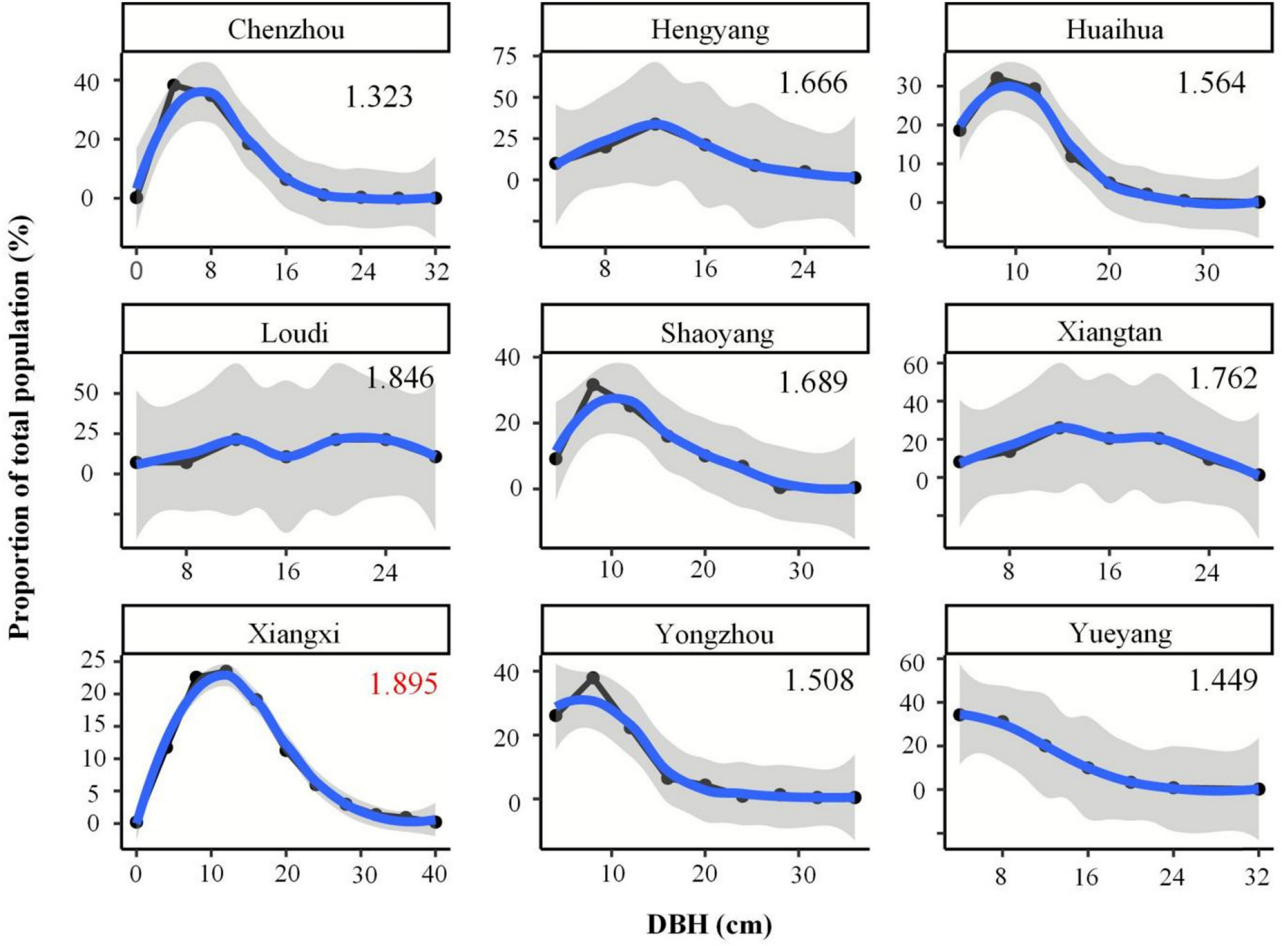
DBH distributions for nine cities (Data points represent the percent trees in each city within the DBH class. Shannon–Wiener index of each city is in the upper right corner. Blue line represents the fitting curve, at 95% confidence).

The average age of each plot was 24, which indicated that the forest was in the middle age. The distributions of DBH class across the nine cities are presented in Figure 3 with binary polynomial fitting curve and Shannon–Wiener index (also in Table 2). An exponential reduction in the proportion of trees in each subsequently larger diameter class was found in the Chenzhou, Huaihua, Shaoyang, Xiangxi, and Yongzhou cities. These cities had a large proportion of trees with small DBH (<15 cm). Hengyang, Xiangtan, and Yueyang cities showed a linear decrease in the proportion of trees in each subsequently larger diameter class. Loudi exhibited an even diameter distribution, whereby the largest DBH class had the same proportion with others. Most cities showed a maturing structure. The DBH diversity of Xiangxi city was the highest (1.895) followed by Loudi city (1.846), which indicated a better DBH structure. The DBH diversity of Chenzhou and Yueyang city was 1.323 and 1.449, respectively, which indicates the presence of a relatively simple stand structure, and needs more effort to promote the forest structure.

### 3.3 Driving factors of DBH diversity

**Figure 4:**
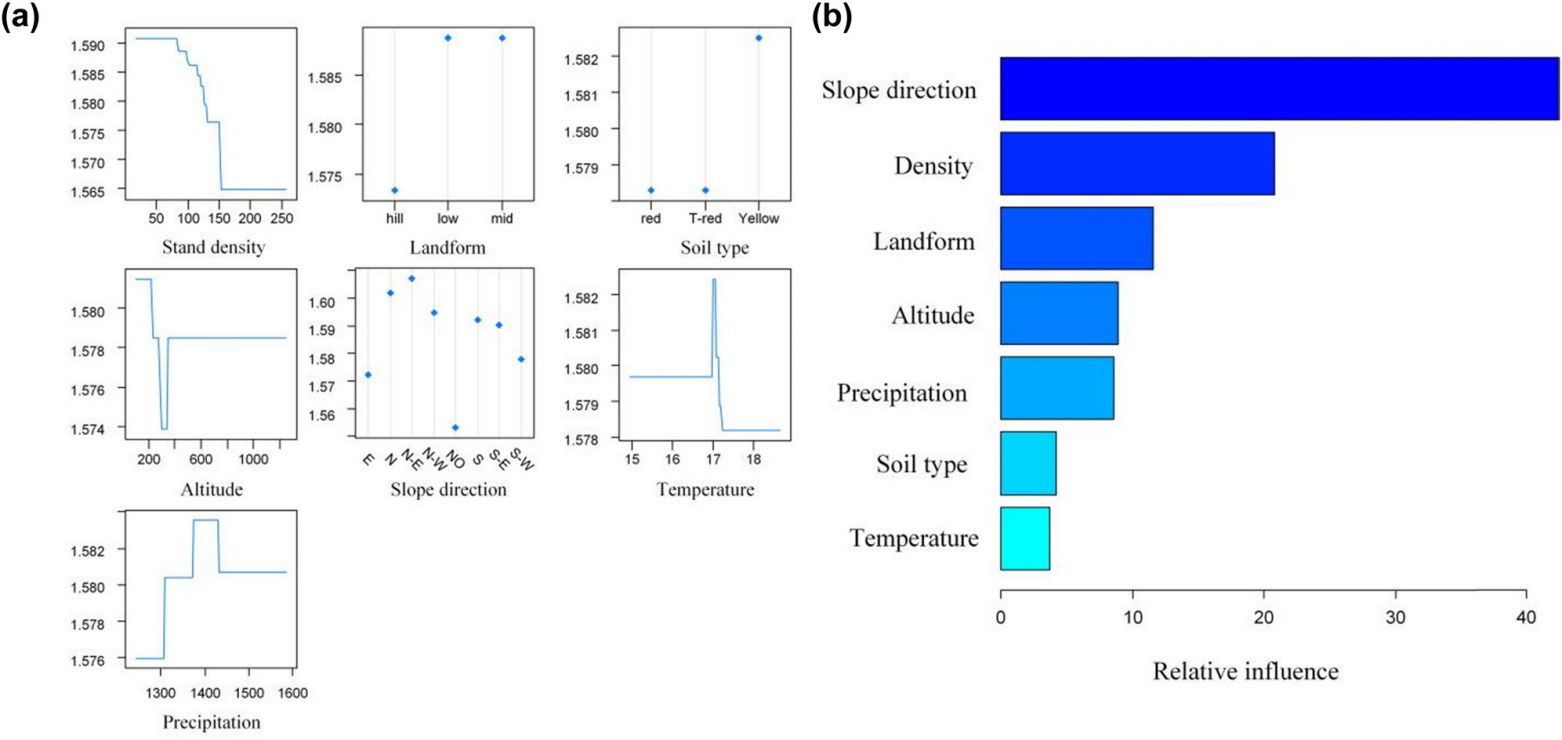
Relationships between the DBH diversity and seven factors: (a) effect of single factor and (b) importance of factors. Vertical (*y*) axis represents the DBH diversity index.

The effect of each single factor is shown in Figure 4a. DBH diversity sharply decreased, when the stand density reached 150 (per 1,000 m^2^). In case of the landform, the plots in low- and mid-mountain exhibited a higher DBH diversity. Considering the soil type, the plots with yellow soil exhibited a higher DBH diversity. In case of the altitude factor, the DBH diversity increased at 200 m and then decreased and at last remains unchanged at altitude over 400 m. In case of the landform and slope direction, the plots with north, north-east, and north-west presented a higher DBH diversity; but the plots with lateritic and yellow soil exhibited a higher DBH diversity. Considering the temperature factor, the plot with 17℃ showed a highest DBH diversity, and in case of the precipitation, the plot with precipitation over 1,500 mm exhibited a higher DBH diversity.

**Figure 5:**
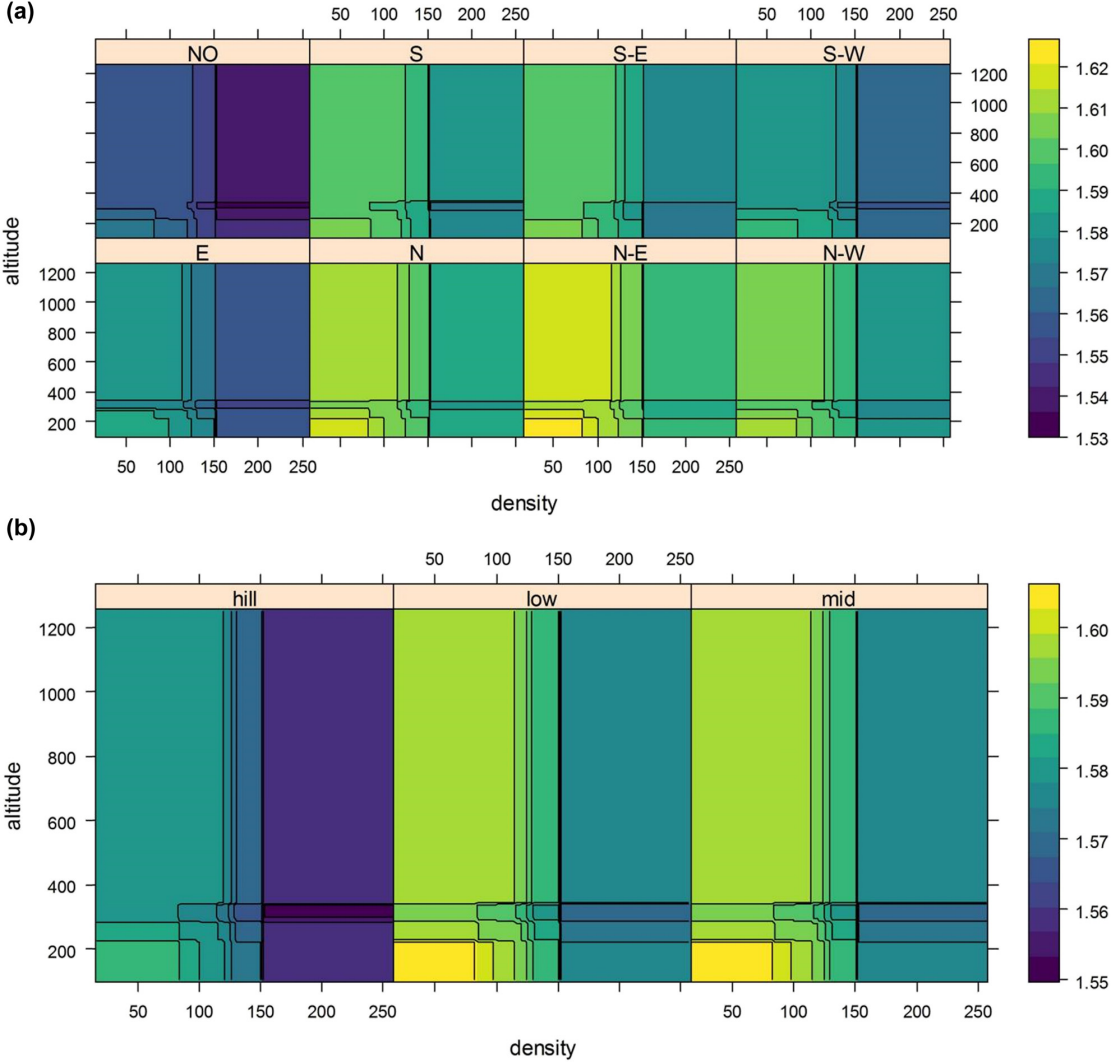
Three-way plots of marginal effect: (a) stand density, altitude, and slope direction on DBH diversity and (b) stand density, altitude, and landform on DBH diversity (color represents the value of DBH diversity).

The importance of seven factors is presented in Figure 4b. The diversity was most affected by slope direction (over 40% relative influence), followed by stand density, landform, altitude, precipitation, soil type, and temperature. Additionally, the three-way marginal analysis was conducted. Figure 5a presents the three-way margin plot of the stand density, altitude, and slope direction. The DBH diversity reached the maxima at an altitude below about 220 m and stand density below 80 (per 1,000 m^2^), while it was minimum at an altitude between 200 and 400 m and stand density over 150 (per 1,000 m^2^). It is notable that the DBH diversity of the plot with south-east was minimum at an altitude below 380 m. In Figure 5b (three-way margin plot of stand density, altitude, and landform), it can be seen that the DBH diversity reached the maximum value at an altitude below 220 m and stand density below 80 (per 1,000 m^2^), and was minimum at an altitude between 300 and 370 m and stand density over 150 (per 1,000 m^2^).

### 3.4 Vertical and crown structure analysis

Using the TSTRAT method, a preliminary analysis showed that 13 plots were characterized by two vertical layers, and 19 were characterized by one vertical layer. The dominant layer represented 94.74% of the trees, the average tree height of dominant layer was 9.18 m, the average crown length was 4.73 m, and the average DBH was 11.94 cm. Specifically, in the plots characterized by two canopy layers, the average percentage of dominant layer was 84.64%. This analysis of the vertical structure showed a reduced complexity of the stand, which may cause competition between the trees in the dominant layer.

Path analysis of the crown width showed that the degree of direct effect was higher (higher absolute value of coefficient), and both DBH and tree height had significant effects on the crown width (Table 3, *p* < 0.001). DBH showed a significant positive direct effect on the crown width in all stages, and the effect reached the peak at mature stage (1.03). The direct effect of DBH was higher than that of tree height. The tree height exhibited a positive direct effect on the crown width, except for the mature stage (*p* < 0.001), and presented a direct negative effect on the crown width in the mature stage (−0.69). The result showed that DBH estimation was more accurate in quickly obtaining the crown width of all stages.

**Table 3:** Path analysis coefficient of DBH and tree height to crown width

Forest age stage	Direct path coefficient		Indirect path coefficient	
	DBH	Tree height	DBH	Tree height
Young aged	0.47***	0.27***	0.32	0.17
Mid aged	0.42***	0.17***	0.23	0.08
Near-mature	0.48***	0.30***	0.35	0.20
Mature	1.03***	−0.69***	0.52	−0.06

Note: ‘***’ represent the significant level of 0.001.

Path analysis of the live crown ratio showed that the degree of direct effect was higher (higher absolute value of coefficient). DBH showed a significant positive effect in the mid- and near-mature stages, while had significant negative effects in the mature stages (Table 4, *p* < 0.001). The tree height had a significant positive effect in the young and mature stage, and had a significant negative effect in the mid- and near-mature stage (*p* < 0.001). The absolute value of the direct path coefficient of tree height was higher than DBH in the young, mid, and mature stages. The results showed that the tree height estimation was more accurate in quickly obtaining the live crown ratio of the young, mid, and mature stages, while the estimation of DBH was more accurate in near-mature stages.

**Table 4:** Path analysis coefficient of DBH and tree height to live crown ratio

Forest age stage	Direct path coefficient		Indirect path coefficient	
	DBH	Tree height	DBH	Tree height
Young aged	−0.04	0.64***	−0.02	0.39
Mid aged	0.30***	−0.56***	−0.03	0.19
Near-mature	0.42***	−0.15***	0.13	−0.03
Mature	−0.46***	0.47***	0.05	0.06

Note: ‘***’ represent the significant level of 0.001.

## 4 Discussion

Many of the earlier studies on *P. massoniana* forest structures were based on the conditions of few study plots only and/or were situated over a limited GPS range [39–42]. The present study was conducted based on the latest inventory data (2019) of nine cities in the Hunan province with a range of different site conditions, which provided a comprehensive understanding of pure *P. massoniana* forest structure. The results indicated that the distribution of DBH, height, crown width, and crown height in the different stages suggested an unstable state; specifically, the DBH, crown width, and crown length between the succession age stages were small. Due to the early uniform standards of planting and management strategy, most pure *P. massoniana* forests were composed of individuals with same age, and the planting density did not match the stand dynamics, which caused a simple horizontal structure. The DBH fitting distribution curve of Chenzhou, Huaihua, Shaoyang, and Xiangxi in the present study was consistent with a maturing distribution. These cities were rich in forest resource in the Hunan province and had a greater proportion of trees with 5–20 cm DBH. Among the nine cities, Loudi city presented a mature distribution. Yongzhou and Yueyang cities exhibited a youthful distribution, which suggest a recent increase in tree planting efforts [11]. In the DBH distribution, most of the cities were dominated by small trees. In fact, the forest in Loudi that was identified as mature population also indicated that there was a need of more effective management efforts. A close-to-nature management has been applied on many species including the *P. massoniana*, and this strategy simulates the process in a natural forest. A youthful and maturing distribution may help to ensure stability, because the high proportion of seedlings and saplings are recruited into larger diameter classes over time to counteract the high rates of mortality [15]. Previous studies show that a proper introduction of broad-leaved trees into pure *P. massoniana* forests improved the DBH growth and ecological value [43].

There are many ways to quantify structural diversity [44]. Quantify stand structure diversity with tree-size variability were applied in Canada [45]. Size-density indices were used in the contrast between even-aged vs uneven-aged stands [46,47]. DBH is among the most commonly collected data, and is useful in making the management and planning decisions [48]. The diversity characterized by DBH has been used as an indicator of forest stability and ecosystem services [11,15]. However, the extent and the influencing factors of the diversity were rarely studied. In this research, we verified and compared the contributions of the explanatory variables to the DBH diversity. GBM and marginal effect analyses showed that the slope direction strongly influenced the diversity, followed by the stand density, landform, and altitude. In the study area, there was a little difference in the regional climate; hence, the temperature and precipitation were not the primary affecting factors. Slope direction is the most important factor in landform that can affect the species component and production [49]. The solar radiation and angle between the wind direction are different in each slope direction, and the redistribution of water and heat conditions forms different micro-climates on the slope surface in different slope directions [50]. In Hunan province, most forest lies in the mountainous region. The micro-environment caused by slope direction and landform plays a great role in the mountain ecology, and can largely reflect the site quality. Previous studies found that the slope with north direction has a higher content of soil nutrients in the Mediterranean region and Qilian Mountains [51,52]. The higher content of soil nutrition reduces the competition by providing more natural resource. In this research, the DBH diversity was higher in the south direction, indicating that the slope with south direction provided more resource, contradicting the results of the previous study. Furthermore, it was found that the DBH diversity was also affected by other factors according to marginal analysis, such as stand density and altitude. The practical significance of the study shows that the capacity of the land resources should be fully considered during the formulation of a management plan. Thus, in the silviculture and management of *P. massoniana* in Hunan province, the stand density needs to be controlled, and the stand dynamics should be matched for a better DBH structure. According to our analysis, the matching of the suitable stand density the different stand dynamics was proposed. For example, the hill area with altitude over about 200 m should have the stand density below 120 (per 1,000 m^2^), the area with east slope direction and altitude below 300 m should have the stand density below 100 (per 1,000 m^2^). Chinese professional standard-technology regulations of the intermediate and management on *P. massoniana* (LYT2697-2016 suggests that the stand density should be ranged from 97 to 150 (per 1,000 m^2^) in a middle age forest, and the recommended stand density was similar to our findings. Our study supports the national standard-technology to a certain extent, and we proposed preliminary suggestions for the regional forest management. For the cities with poor DBH structure such as Chenzhou and Yueyang, a reasonable density adjustment can be the most simple and effective method.

Tree crown plays an important role in the growth of trees, and it can affect the growth and survival of trees [53], determines the size of the tree [54], and reflects the long-term competition level of the trees [55]. The tree with a bigger crown has a higher photosynthetic capacity, but the respiration of other organs gets enhanced and leads to a low production efficiency [56]. The relationship is that, the smaller the crown area per unit volume, the lower the productivity [57]. In the forest with high stand density, an increased competition between the individual trees has a negative influence on crown width [58], and narrower crowns have been associated with higher stand densities [59]. The crown of young trees is vulnerable to natural disasters, but not the stand density. In this study, the distribution of crown width was narrow, and the multimodal distribution could be caused by natural damage. Additionally, the crown width increased from the middle aged stage to a near-mature stage, and was steady in the mature stage. The crown length showed opposite trend, which indicated the adjustment in the growth strategy of the trees. The vertical structure characterized by TSTRAT indicated that the crown layer appeared quite compact with a small number of gaps. An ongoing competition between the trees in the dominant layer indicated a limited potential diversity, since the vertical distribution of the crown interferes with the presence of suitable habitat to accommodate different plant and animal species. For example, the complex crown structure has a higher bird diversity [60,61].

In this research, the crown width of each age stage had a strong relation with the DBH and tree height, which was consistent with the findings of previous studies [20–22]. Except for the no significant correlation between the live crown ratio and DBH of young aged forest, live crown ratio had a strong connection with DBH and tree height. Since there is a little competition in young aged forest, we assumed that the crown height may be decided by its inherited characteristics. Path analysis showed that the crown width exhibited a significant positive correlation with DBH in each stage, but showed a significant negative correlation with height in the mature stage. These results indicate that the trees tend to increase the height and DBH for the light resource and support, but the wider crown increases the risk of wind-breakage in the mature stage. From the results of live crown ratio and distribution, we can assume that the trees tend to increase the crown width and reduce the crown height in the competition of light and space before maturity, and then a narrow and deep crown gets developed to increase the area of photosynthesis and reduce the risk of wind-breakage in the mature stage.

## 5 Conclusion

In the present study, we presented the diameter and crown structure of pure *P. massoniana* forest in the Hunan province. It was found that the distribution of DBH, height, crown width, and crown height in different stages followed a non-normal distribution. There were statistically significant differences in the DBH distributions across the cities, indicating the apparent qualitative differences. The results indicate that there is a need to undertake measures to improve the forest by the local forest department. Furthermore, the slope direction, landform, and stand density had a stronger effect on the diameter diversity, and some management activities were suggested to use to improve the structure. The relationship between the crown structure and DBH/tree height showed an obvious change according to the different age stages, which highlighted the competition strategy of the population. Our study is the first comprehensive study on the non-spatial structure of pure *P. massoniana* forest in Hunan province, and can provide valuable information in the forest management, planning, and valuation of the ecosystem services.
